# Case Report: Successful Treatment of a Child With COVID-19 Reinfection-Induced Fulminant Myocarditis by Cytokine-Adsorbing *oXiris*® Hemofilter Continuous Veno-Venous Hemofiltration and Extracorporeal Membrane Oxygenation

**DOI:** 10.3389/fped.2022.946547

**Published:** 2022-07-12

**Authors:** Phuc H. Phan, Dung T. Nguyen, Nam H. Dao, Ha T. T. Nguyen, An V. Vu, Son T. Hoang, Lam V. Nguyen, Tung V. Cao, Dien M. Tran

**Affiliations:** ^1^Pediatric COVID-19 Intensive Care Unit, Vietnam National Children's Hospital, Hanoi, Vietnam; ^2^Cardiovascular Center, Vietnam National Children's Hospital, Hanoi, Vietnam

**Keywords:** COVID-19, fulminant myocarditis, *oXiris*® hemofilter, ECMO, case report

## Abstract

**Background:**

Indirect cardiomyocyte damage-related hyperinflammatory response is one of the key mechanisms in COVID-19-induced fulminant myocarditis. In addition to the clinical benefit of using cytokines absorption hemofiltration, the effectiveness of instituting veno-arterial extracorporeal membrane oxygenation (VA-ECMO) support for cardiac compromise has been reported. However, current literature enunciates a paucity of available data on the effectiveness of these novel modalities.

**Case Presentation:**

We reported a 9-year-old boy with recurrent COVID-19 infection-causing fulminant myocarditis, who was treated successfully by using novel modalities of *oXiris*^®^ hemofilter continuous venovenous hemofiltration (CVVH) and VA-ECMO. The patient made a full recovery without any sequelae.

**Conclusion:**

We conclude that the novel highly-absorptive hemofilter CVVH and VA-ECMO may be effective treatment modalities in managing SARS-CoV-2-induced fulminant myocarditis. Our report highlights the need for further well-designed investigations to confirm this extrapolation.

## Introduction

The outbreak of COVID-19 appeared as an extremely contagious illness, which is known to involve multiple organ systems, including respiratory, cardiovascular, thrombotic, gastrointestinal, renal, and neurological manifestations (1). Among them, myocarditis is an uncommon presentation with an estimated incidence below two percent and variably from mild to fulminant myocarditis (2, 3). In general, though pediatric cases are usually reported as asymptomatic or mild scenarios compared to adults, there is still scarce data about the prevalence and outcome of pediatric COVID-19-induced myocarditis (4).

The pathophysiology of COVID-19 infection is initiated by binding the viral spike (S) protein to the cellular angiotensin-converting enzyme 2 (ACE2) receptors, facilitating SARS-CoV-2 entries into host cells. ACE2 expression has been detected in multiple organ systems, including cardiomyocytes, consistent with direct viral cardiotoxicity (5). Another scholar described the “cytokine storm” as the hypothesized pathophysiology of COVID-19-induced myocarditis, similar to a massive release of the pro-inflammatory cytokines in severe COVID-19 patients (5, 6). Remarkably, interleukin-6 (IL-6) plays an orchestrator role in the inflammatory response and acts extensively in T-lymphocyte activation, leading to positive feedback from the immune system and myocardial damage (7).

Based on the aforementioned pathophysiology, the efficacy of different comprehensive treatments of myocarditis-associated COVID-19, such as antiviral drugs, corticosteroids, immunotherapeutic, and supportive extracorporeal methods, is being investigated (8). To date, continuous renal replacement therapy (CRRT) with a highly cytokine and endotoxin adsorptive membrane (*oXiris*^®^)-an adjuvant therapy for controlling the hyperinflammatory syndrome in sepsis and other serious illnesses, has been applied to severe COVID-19 patients (9). Also, the role of veno-arterial extracorporeal membrane oxygenation (VA-ECMO) in supporting cardiovascular collapse–linked to COVID has been documented (2). However, the existing literature described mainly just case by case, and solid evidence on optimizing the management of such cases is required.

Here, we report a 9-year-old boy with COVID-19 reinfection who developed progressive cardiac deterioration. He was diagnosed with SARS-CoV-2-induced fulminant myocarditis and was treated successfully by using novel modalities of *oXiris*^®^ hemofilter-continuous venovenous haemofiltration (CVVH) and VA-ECMO. This report contributes to the current pediatric literature on COVID-19-induced myocarditis and highlights the need for further investigations in determining the effectiveness of these supportive extracorporeal treatment modalities in such cases.

## Case Report

A previously healthy 9-year-old boy suffered from a high-grade fever (39°C) and dry cough without dyspnea on 18 February 2022. Because the child had close contact with a confirmed COVID-19 case, he was then tested for SARS-CoV-2 detection in nasopharyngeal swab by using a rapid antigen test, which showed a strongly positive result. These symptoms discontinued after 5 days, not requiring hospitalization, and the antigen test returned negative on 23 February 2022.

On 12 March 2022, the child got a sore throat, massively dry cough, and high non-intermittent fever. He did not have any rashes, vomiting, or diarrhea. After 2 days, he was admitted to a local hospital, where the real-time polymerase chain reaction (RT-PCR) of his nasopharyngeal specimen confirmed the presence of SARS-CoV-2 RNA. After 2 days, he developed chest pain, fatigue, and progressively dyspnea requiring supplemental oxygen by a face mask. For the rapid worsening of his clinical presentations, the patient was then transferred to Vietnam National Children's Hospital on the 4th day of his current illness.

On admission to our COVID-19 Intensive Care Unit, the patient appeared agitation and was on cardiac deterioration with invasive blood pressure (IBP) of 90/54 mmHg, non-gallop tachycardia of 160 beats per minute (bpm), and cool extremities with delayed capillary refill time (CRT) of 3 s. His shortness of breath on exertion required immediate endotracheal intubation. He had good air entry to both lungs and no crackle on respiratory examination. Hepatosplenomegaly sign or peripheral edema was absent. The electrocardiogram demonstrated a sinus tachycardia and diffuse ST elevation without reciprocal changes. His Pediatric Risk of Mortality III (PRISM-III) score was 11.

**Figure 1:**
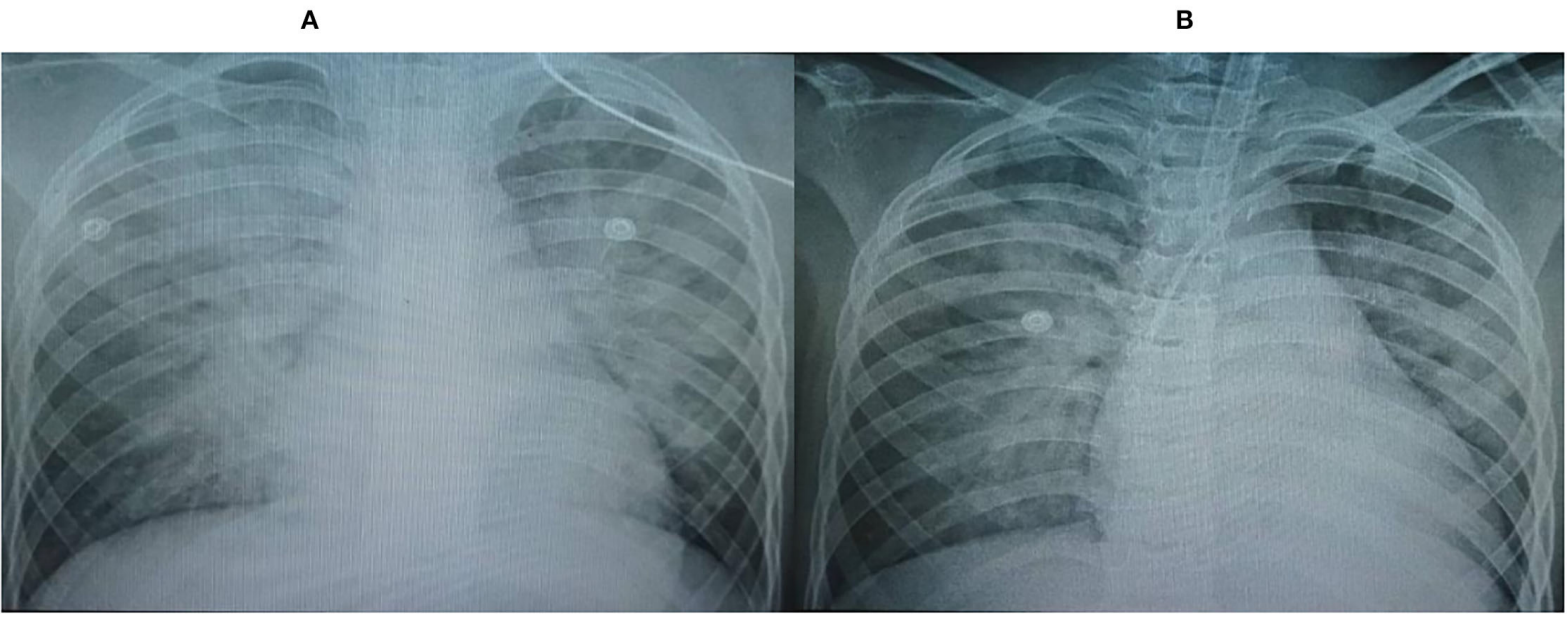
Chest x-rays (CXRs) revealed pulmonary opacification of the bilateral perihilar region and cardiomegaly with a mildly increased cardiothoracic ratio. **(A)** On admission, **(B)** Post-24 h hemofiltration.

The chest x-rays (CXRs) revealed pulmonary opacification of the bilateral perihilar regions and cardiomegaly with a mildly increased cardiothoracic ratio (Figure 1A). Two-dimensional (2-D) color Doppler echocardiography demonstrated a dilated left ventricle (left ventricular diastolic diameter at 43.2 mm) with diffuse hypokinesia and severe systolic dysfunction (left ventricular ejection fraction (LVEF) at 30%), and mild bicuspid regurgitation. The findings of pericardial effusion or coronary abnormalities were absent.

Arterial blood gas (ABG) analysis showed pH of 7.38, partial pressure of oxygen (PaO_2_) of 89 mmHg, partial pressure of carbon dioxide (PaCO_2_) of 46 mmHg, bicarbonate (${\text{HCO}}_{3}^{-}$) of 27.8 mmol/l, oxygen saturation of 99% with FiO_2_ of 0.75, and lactate of 1.2 mmol/l. The mixed venous oxygen saturation (ScvO_2_) was 66%.

Blood tests revealed a remarkable elevation in cardiac troponin I (Trop I) (18.99 μg/l, normal <0.05 μg/l), elevated N-terminal pro-brain natriuretic peptide (NT-proBNP) (1,329 pg/ml, normal <50 pg/ml), normal Creatine Kinase-Myocardial Band (CK-MB) level (37.3 U/l, normal <24 U/l), slight increase in C-reactive protein (CRP) level (37.3 mg/l, normal <6 mg/l), elevated lactate dehydrogenase (LDH) (300 U/l, normal <266 U/l), elevated ferritin level (904 ng/ml, normal <140 ng/ml), and elevated D-dimer level (1,171 ng/ml, normal <500 ng/ml), slightly elevated IL-6 (7.06 pg/ml, normal <6 pg/ml). Other laboratory investigations were unremarkable as following: neutrophils (NEU) count of 5,540 cells/μ, lymphocytes (LYM) count of 1,160 cells/μl, hemoglobin (HGB) of 12.3 g/dl, platelets (PLT) count of 274 × 10^3^ cells/μl, prothrombin time (PT) of 12.8 s, activated partial thromboplastin time (APTT) of 33 s, fibrinogen of 3.9 g/l, blood urea nitrogen of 4.29 mmol/l, creatinine of 49.9 μmol/l, aspartate aminotransferase of 167 U/l, alanine aminotransferase of 36 U/l, blood glucose level of 8.4 mmol/l, sodium of 134 mmol/l, potassium of 3.5 mmol/l, chloride of 102 mmol/l, calcemia of 2.08 mmol, and albumin of 33.3 g/l.

With clinical manifestations and detection of SARS-CoV-2 by RT-PCR, the child's condition was consistent with cardiogenic shock complicated by acute COVID-19 infection-induced fulminant myocarditis. He was initially managed by positive pressure mechanical ventilation support and continuous administration of dobutamine (15 μg/kg/min), nor-epinephrine (0.1 μg/kg/min), and milrinone (0.5 μg/kg/min). The patient also received a dexamethasone dose of 0.3 mg/kg once daily and empiric ceftriaxone for concern of superimposed bacterial infection. He stabilized for 4 h prior to developing progressive cardiovascular collapse, which was unresponsive to the high-dose inotropic infusion with dobutamine, nor-epinephrin, and milrinone dose of 20 μg/kg/min, 0.4 μg/kg/min, and 0.5 μg/kg/min, respectively. His heart rate was 170 bpm, and his IBP remained low at 90/42 mmHg.

We initiated CVVH with a specialized hemofilter composed of AN69 and polyethyleneimine (AN69ST/PEI, *oXiris*^®^, Baxter^®^ France) for increased clearance of cytokines, being consistent with “cytokines storm” as a main pathophysiologic mechanism in COVID-19-induced myocarditis (10). We used the PrismaFlex machine (Gambro, Hechingen, Germany) with a blood flow of 5 ml/kg/min and a replacement of 50 ml/kg/h *via* a double-lumen 11 Fr catheter (Gambro, Hechingen, Germany) inserted into his femoral vein. Initially, no fluid removal was set and then adjusted compatibly with his fluid balance. We targeted the APTT around 60–80 s using unfractionated heparin infusion for systemic anticoagulation. The *oXiris*^®^ hemofilter was replaced every 24 h.

**Figure 2:**
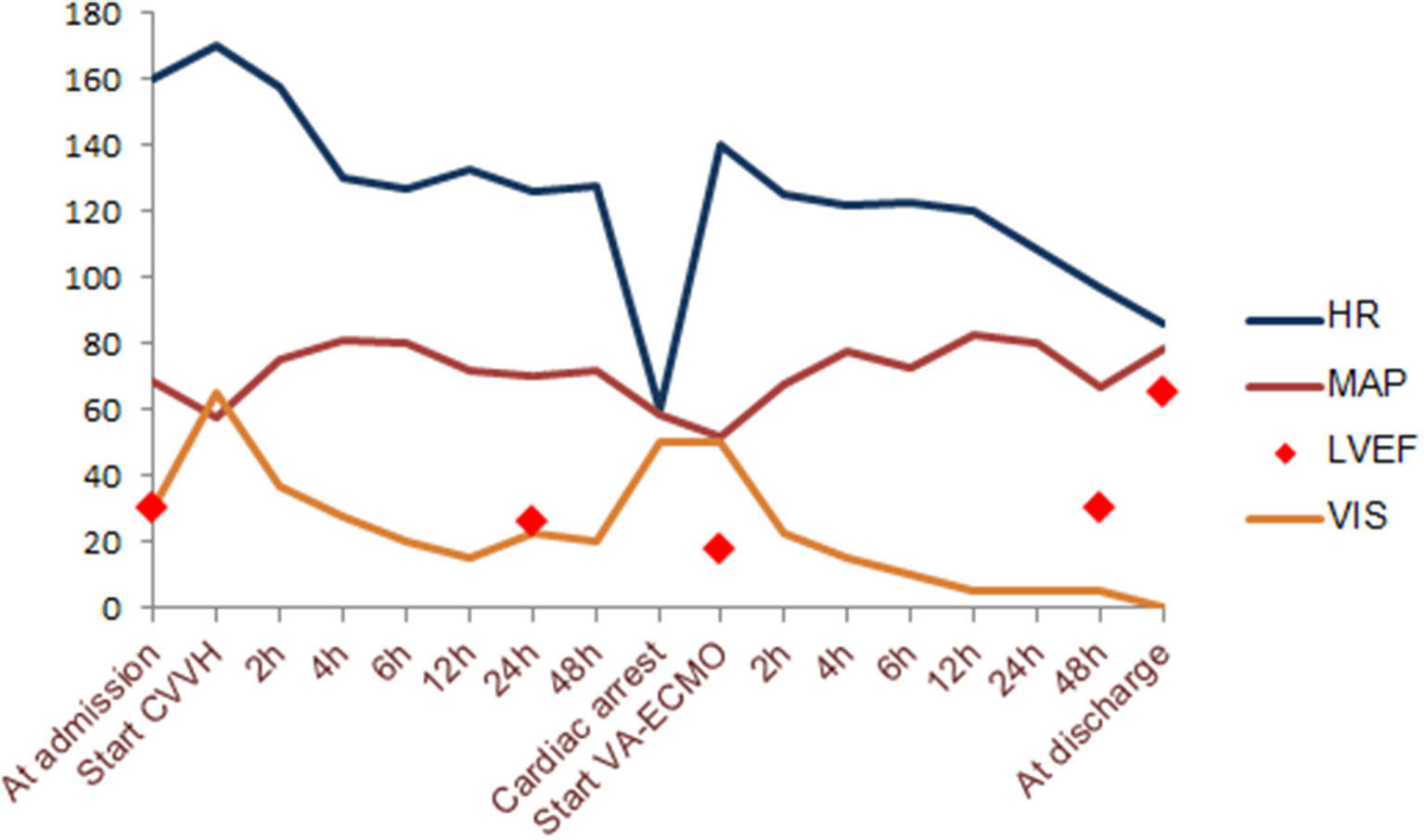
Hemodynamic change according to timeline during supportive extracorporeal treatment modalities. VIS, vasoactive-inotropic score; LVEF, left ventricular ejection fraction; MAP, mean arterial pressure; HR, heart rate.

After 6 h of CVVH, the child's clinical condition improved significantly (Figure 2). His heart rate gradually decreased to 127 bpm, and his IBP was 110/65 mmHg. We tapered the dose of nor-epinephrine and dobutamine infusion and ceased after 5 and 10 h following CVVH, respectively. His post-24-h PRISM-III score was 6. Although the LVEF at post-24-h echocardiography remained poor at around 24%, markers of myocardial injury showed a significant decline (Table 1). All inflammatory markers showed no progressive elevation, including CRP, procalcitonin, LDH, ferritin, fibrinogen, IL-6, and D-Dimer (Table 1). In addition, the post-24-h CXRs showed improvement in lung opacity (Figure 1B). The *oXiris*^®^ hemofilter CVVH was discontinued after a total of 72 h.

**Table 1:** Laboratory results.

**Parameters**	**Admission**	**24 h post hemofiltration**	**VA-ECMO**	**D4**	**D10**	**D12**	**D15**	**Discharge**	**2-week after discharge**
WBC (cells/μ)	6,930	10,230	10,060	7,800	10,490	12,410	12,990	6,840	7,910
NEU (cells/μ)	5,540	8,510	8,390	5,550	4,480	9,600	8,390	3,490	4,810
LYM (cells/μ)	1,160	890	850	1,620	3,920	1,450	3,330	2,340	1,880
PLT (× 10^3^ cells/μ)	220	234	185	172	117	70	321	795	361
Ferritin (ng/mL)	904	591	336	535	NT	470	NT	NT	322
LDH (U/L)	300	316	589	280	272	345	NT	NT	NT
IL-6 (pg/mL)	5.34	14.5	NT	NT	NT	NT	NT	NT	NT
D-dimer (ngFEU/mL)	1,171	282	300	420	1,630	40,534	10,149	2,340	1,318
CRP (mg/L)	37.3	11.45	10.3	11.2	38	12.1	10.5	NT	1.95
CK-MB (U/L)	37.3	26.7	26.7	11.2	9.4	13.3	31.7	21.7	17.2
Pro-BNP (pgm/L)	1,329	979	984	721	369	262	445	73.7	23
Troponin I (μg/L)	18.99	9.13	9.21	3.39	0.26	0.1	0.08	0.056	0.031
LVEF (%)	30	24	18	30	48	55	61	65	49
COVID-19 RT-PCR Ct (copies/mL)	27						37		

*WBC, white blood cell; NEU, neutrophils; LYM, lymphocyte; PLT, platelets; LDH, lactate dehydrogenase; IL-6, interleukin-6; CRP, C-reactive protein; CK-MB, creatine kinase-myoglobin binding; Pro-BNP, N-terminal pro b-type natriuretic peptide; LVEF, left ventricular ejection fraction.; RT-PCR Ct, Reverse transcription polymerase chain reaction cycle threshold; NT, not tested*.

Twenty-two hours after discontinuing the absorptive filter, the child suddenly deteriorated with his heart rate of <60 bpm, and his IBP of 88/45 mmHg. The return of spontaneous circulation was achieved after 30 s of conventional cardiopulmonary resuscitation. However, his condition deteriorated drastically, though high-dose inotropic agents were administrated with dobutamine (15 μg/kg/min), epinephrine (0.2 μg/kg/min), nor-epinephrine (0.1 μg/kg/min), and milrinone (0.5 μg/kg/min). Follow-up echocardiography witnessed a global hypokinesis with poorly impaired LVEF estimated at 18%. The patient was immediately managed with VA-ECMO support. We successfully cannulated the patient *via* the right internal jugular vein using a 20-F drainage cannula and the right internal carotid artery using a 17-F perfusion cannula. During VA-ECMO, unfractionated heparin was continuously administrated to achieve an APTT of 60–80 s. The initial flow rate was set as 2.2l/min and gradually reduced according to his improved hemodynamic condition. The patient remained on dexamethasone therapy of 0.3 mg/kg daily until hospital day 6. Daily echocardiography suggested gradually recovered cardiac function with the progressive LVEF change, as shown in Figure 2. By LVEF of 50%, VA-ECMO was successfully decannulated on hospital day 12, and the patient was extubated successfully on hospital day 15. Echocardiography just before discharge estimated LVEF of 65%, requiring further long-term follow-up. He was discharged home on the 28th day after admission without any sequelae.

His microbiology investigations failed to detect mycoplasma pneumonia, B hepatitis virus, Epstein–Barr virus, cytomegalovirus, human herpesvirus-6, human immunodeficiency virus, adenovirus, and enterovirus. His COVID-19 RT-PCR of endotracheal specimens continued to be positive until hospital day 18.

At the 2-week follow-up after discharge, except for a mild reduction of his LVEF at 49% demanding regular follow-up, his clinical condition and re-examination tests were normal (Table 1).

## Discussion

During robust data on COVID-19, acute myocarditis has been reported to be associated with COVID-19 infection in adults (5, 11). However, current findings on the prevalence and outcomes of this manifestation are sparse, particularly in the pediatric population, with a limited number of case reports (3, 10). We reported a 9-year-old boy with COVID-19-induced fulminant myocarditis presenting as progressively cardiovascular collapse with predicted high mortality. To the best of our knowledge, this is the first report describing successful treatment using highly absorptive CVVH and VA-ECMO modalities.

The COVID-19 reinfection presents one peculiarity in our case report. Previous data revealed that reinfections after primary SARS-CoV-2 infection are uncommon, and the reinfection risk in children is remarkably lower than in adults (12). This finding persisted with the insight into children's ability to more robust humoral and cellular responses to SARS-CoV-2, potentially longer immunity sustained beyond 12 months, and evidence of cross-protection against new SARS-CoV-2 variants (13). However, reinfection hazard also depends on the difference between SARS-CoV-2 variants over time, possibly consistent with our case during a new wave of the Omicron BA.2 variant by mid-March 2022 in Vietnam. Unfortunately, published data on the severity and outcome of SARS-CoV-2 reinfections in children remained poorly and controversial. Nguyen et al. reported no significant differences in hospitalization or severity between the first and second infections (14). Similarly, another national study on children reinforces that reinfections were not linked to the risk of severity regarding hospitalization or intensive care admissions and 28-day fatality (12). In contrast, according to Wang et al. SARS-CoV-2 reinfections are seemly more severe than the primary one; since a second infection by a different variant could arouse total immunity, which may not be achieved on the previous infection (15, 16). Another peculiarity is that our patient's symptoms initiated after more than 2 weeks of his first COVID-19 infection, comply with a post-infectious, immunological consequence after 2–4 weeks of SARS-CoV-2 exposure, the multisystem inflammatory syndrome in children (MIS-C) (17). His SARS-CoV-2-specific antibodies against the nucleocapsid (N) and the spike (S) protein were positive, at 5.53 and 21.88 mg/ml, respectively. Thus, these overlapping clinical manifestations of cardiac complications could lead to a misclassification bias between COVID-19-myocarditis and cardiovascular involvements related to MIS-C. Of note, cardiac features of MIS-C are common; specifically, left ventricular dysfunction was revealed in 35–100% of children (18). The laboratory findings of our case showed elevations of D-dimer, CRP, ferritin, LDH, and lymphocytopenia, all indicating inflammatory responses as seen in MIS-C. However, the usual clinical presentations in MIS-C include rash, bilateral non-purulent conjunctivitis or muco-cutaneous inflammation signs, and gastrointestinal symptoms (19) were absent in our case. Moreover, patients with MIS-C were found to more rapidly normalize in LVEF within 1–2 weeks, regarding LV dysfunction-induced severe systemic inflammation and acute stress, unlike ischemia or viral myocarditis (20). Our patient required a 4-week-interval to make a full recovery of LVEF.

The probable pathophysiology in SARS-CoV-2-induced myocarditis is likely similar to that of other viruses, including direct myocardial damage *via* binding to ACE2 and indirect myocardial toxicity by an overwhelming immune-inflammatory response (21). Additionally, the acute hyper-inflammatory response develops hypercoagulability leading to acute myocardial ischemia (22). To date, there is still a lack of data identifying the cytokinemia profile in SARS-CoV-2-mediated myocarditis, though several reports revealed the elevation of proinflammatory cytokines, including IL-6, IL-1β, IL-2, IL-8, IL-17, Tumor Necrosis Factor (TNF), granulocyte - colony stimulating factor (G-CSF), Granulocyte macrophage - colony stimulating factor (GM-CSF), IFN-gamma-inducible protein 10 (IP10), monocyte chemoattractant protein 1 (MCP1), and Macrophage inflammatory protein 1 alpha (MIP1α)… (23–26). Hence, CRRT using the *oXiris*^®^ hemofilter, a highly permeable membrane with enhanced property for endotoxin and cytokine removal, may be applied in fatal SARS-CoV-2-induced hypercytokinemia (26). The *oXiris*^®^ hemofilter showed effective untargeted removal of 27 pro- and anti-inflammatory mediators in critically ill patients (27). Reducing inflammatory response by using adsorption of cytokines has been widely described in sepsis (27–29). However, the effectiveness of this novel treatment modality in COVID-19- related cytokine storm remains unclear, particularly in the pediatric population with limited data. In adults, successful treatment of critically ill COVID-19-induced fulminant cytokine release by cytokine hemoadsorption was demonstrated but mainly from a handful of case studies, noting improvement in clinical recovery, multiple organ dysfunction scoring systems, coagulopathy, and a reduction in hyperinflammatory (9, 30–32). Contrarily, a single-central study from Kang et al. (33) concluded that CRRT with *oXiris*^®^ hemofilter might not effectively alleviate hypercytokinemia in critical COVID-19 without acute kidney injury. In summary, there is not enough evidence-based data to confirm the effectiveness of hemoadsorption in COVID-19-induced hyperinflammation; therefore, further well-designed investigations on this issue are needed.

While many reports reinforce the key role of IL-6 in exacerbating cardiotoxicity induced by cytokine storm (7, 30, 34), the intriguing question is whether clinical outcomes in COVID-19 are favorable if relatively low levels of circulating IL-6? Of note, Bagate F et al. highlighted the prominent contribution of IL-10, GM-CSF, and IP-10 in the pathology of cardiac dysfunction in COVID-19 (35). The authors also described three serum cytokines profiles with a prominent role of different non-IL-6 cytokines according to LVEF, including hypokinetic patients (LVEF 28–55%), normokinetic patients (LVEF 55–67%), and hyperkinetic patients (LVEF 67–80%) (35). Additionally, serum mediator levels depend on several factors: production rate, the available cell receptors, the clearance of such mediators, and the receptor's affinity to such mediators (29). Unlike TNF-soluble receptors and IL-1 receptors with inhibitory function, the IL-6 receptor is an agonist one; hence a low IL-6 level with a high level of the receptors induces more cellular response than a high level of IL-6 with a low level of receptors. Consequently, a low IL-6 level alone may not be very predictive of the low risk of multiple organ response (29). These findings supported the indication for CRRT with cytokine absorption in our case, despite the low IL-6 level at pre-hemofiltration. Because of unavailable assays measuring non-IL-6 cytokine concentrations, we could not entirely rule out their contribution to myocarditis-induced hypercytokinemia. Therefore, we decided to use the oXitis filter by CVVH modality after carefully considering its effectiveness and adverse effects. After applying CRRT, there was no progressive elevation of all non-specific inflammatory markers, including CRP, procalcitonin, LDH, ferritin, fibrinogen, and D-Dimer, which were universally reported as prognostic markers of severity (24). Thus, it was likely that our patient's clinical condition, lung injury, and biochemical cardiac markers were significantly improved as the benefit of absorptive hemofiltration, though no evidence of cytokines amelioration was shown.

Given the acute respiratory distress syndrome (ARDS) as a prominent feature of COVID-19, the role of veno-venous (V-V) ECMO support is evolving (36). The challenging question is if the benefits of VA-ECMO could outweigh the risks of adverse events to recommend placing VA-ECMO bypass in COVID patients? In adults, the highest-priority VA-ECMO for cardiogenic shock-related SARS-CoV-2 is based on an individual basis and with a multidisciplinary team approach, including the young, the critically ill, and those with the greatest perceived benefit and fewest or no comorbidities, as well as healthcare workers (36). Currently, published extracorporeal life support organization (ELSO) guidelines recommend instituting V-A support for cardiac compromise associated with COVID-19-induced myocarditis and MIS-C, applying standard pediatric institutional ECMO protocols (2). Nonetheless, existing literature surrounding the outcome of VA-ECMO in myocarditis-mediated SARS-CoV-2 in the pediatric population question is limited only case by case and requires ongoing investigations. To the best of our knowledge, our case is the second report in pediatric literature about the application of VA-ECMO in the successful management of SARS-CoV-2-induced fulminant myocarditis. Previously, Buitrago et al. reported using 7-day-course VA-ECMO as a bridge to full recovery on a 12-year-old girl with myocarditis linked to COVID-19 (3).

Our treatment as an integrative therapy approaches two rationales: (1) using an *oXiris*^®^ hemofilter to limit further cardiotoxicity according to possible indirect damage in fulminant hypercytokinemia-associated SARS-CoV-2; (2) timely instituting short-course VA-ECMO support for severely impaired LVEF in expectation of further complete cardiac recovery. However, this report has two limitations: (1) only measuring circulating concentrations of IL-6 due to unavailable assays for other cytokines, (2) the diagnosis of myocarditis merely based on clinical manifestations, 2-D color Doppler echocardiography, and elevated level of cardiac biomarkers, without magnetic resonance imaging or histological evidence.

## Conclusion

We conclude that the novel highly-absorptive hemofilter CVVH and VA-ECMO may be effective treatment modalities in managing fulminant myocarditis-associated SARS-CoV-2. Our report expresses the need for further well-designed investigations to confirm this extrapolation.

## Data Availability Statement

The original contributions presented in the study are included in the article/supplementary material, further inquiries can be directed to the corresponding author/s.

## Ethics Statement

The studies involving human participants were reviewed and approved by Biomedical Research Ethics Committee, Vietnam National Children's Hospital. Written informed consent to participate in this study was provided by the participants' legal guardian/next of kin.

## Author Contributions

PP, TC, and DT provided the concept for the study and were a major contributor to the manuscript revision. DN, ND, AV, LN, and SH were involved in the patient care and collected the data regarding the patient's history and clinical course, as well as the trends in vital parameters. HN collected clinical data, reviewed the literature, and drafted the manuscript. All authors read and approved the final manuscript.

## Conflict of Interest

The authors declare that the research was conducted in the absence of any commercial or financial relationships that could be construed as a potential conflict of interest.

## Publisher's Note

All claims expressed in this article are solely those of the authors and do not necessarily represent those of their affiliated organizations, or those of the publisher, the editors and the reviewers. Any product that may be evaluated in this article, or claim that may be made by its manufacturer, is not guaranteed or endorsed by the publisher.
